# Okadaic acid activates the PKR pathway and induces apoptosis through PKR stimulation in MG63 osteoblast-like cells

**DOI:** 10.3892/ijo.2013.1911

**Published:** 2013-04-17

**Authors:** TATSUJI HANEJI, KANJI HIRASHIMA, JUMPEI TERAMACHI, HIROYUKI MORIMOTO

**Affiliations:** 1 Department of Histology and Oral Histology, Institute of Health Biosciences, The University of Tokushima Graduate School, Kuramoto, Tokushima 770-8504;; 2 Department of Anatomy, School of Medicine, University of Occupational and Environmental Health, Yahatanishi, Kitakyushu 807-8555, Japan

**Keywords:** PKR, IκBα, NF-κB, phosphorylation, dephosphorylation, okadaic acid

## Abstract

Double-stranded RNA-dependent protein kinase (PKR) is one of the players in the cellular antiviral responses and is involved in transcriptional stimulation through activation of NF-κB. Treatment of the human osteosarcoma cell line MG63 with the protein phosphatase inhibitor okadaic acid stimulated the expression and phosphorylation of IκBα, as judged from the results of real-time PCR and western blot analysis. We investigated the functional relationship between PKR and signal transduction of NF-κB by establishing PKR-K/R cells that produced a catalytically inactive mutant of PKR. Phosphorylation of eIF-2α, a substrate of PKR, was not stimulated by okadaic acid in the PKR-K/R cells, whereas okadaic acid induced phosphorylation of eIF-2α in MG63 cells. Phosphorylation of NF-κB in MG63 cells was stimulated by okadaic acid; however, okadaic acid did not induce phosphorylation of NF-κB in the PKR-K/R cells. Finally, okadaic acid-induced apoptosis was inhibited in the PKR-K/R cells. Our results suggest that okadaic acid-induced phosphorylation of IκBα was mediated by PKR kinase activity, thus, indicating the involvement of this kinase in the control mechanism governing the activation of NF-κB and induction of apoptosis.

## Introduction

Double-stranded RNA-dependent protein kinase (PKR) is an abundantly expressed serine/threonine protein kinase which is activated by double-stranded RNA (dsRNA), interferons, cytokines, stress signals, and viral infection (1,2). PKR is also involved in several signal transduction pathways, such as mitogen-activated protein kinase (MAPK), nuclear factor of κB (NF-κB), inhibitor of NF-κB (IκB) and Smad (3–5). PKR is activated through autophosphorylation and once activated the enzyme phosphorylates certain substrates including the α-subunit of eukaryotic initiation factor 2 (eIF-2α) (6,7). The PKR-eIF-2α cascade has been implicated as a general transducer of apoptosis in response to a variety of stimuli (8–12). It was reported that PKR was dephosphorylated by serine/threonine protein phosphatases type 1 (PP1) (13,14). PP1 binds directly to PKR and reduces dsRNA-mediated auto-activation of PKR (15). PP1 may regulate the activities of both PKR and eIF-2α by dephosphorylating them and thus might block the protein synthesis and apoptosis.

Apoptosis is one of the essential steps in the maintenance of normal cell populations of adult mammals and occurs continually in various cell populations. Apoptosis is a morphologically and biochemically distinct mode of cell death that plays major roles during embryogenesis, carcinogenesis, cancer treatment, or immune and toxic cell killing (16–20). The cytological apparent stages of apoptosis are rapid condensation of chromatin and fragmentation of the cells with membrane-enclosed apoptotic bodies that are phagocytosed and digested by nearby resident cells (21). A biochemical characteristic feature of the process is double-strand cleavage of nuclear DNA at the linker regions between nucleosomes, leading to the production of oligonucleosomal fragments with 180–200 bp, which results in a characteristic laddering pattern on agarose gel electrophoresis (22,23).

Okadaic acid (OA) is a toxic polyether fatty acid produced by several dinoflagellates and is a potent inhibitor of PP1 and PP2A. The use of this agent has led to the understanding that the phosphorylation and dephosphorylation status is related to cellular regulation, including the biological end-point, apoptosis (24–26). We previously reported that OA induced apoptosis in human osteoblastic cells (27–29). Protein kinases and phosphatases were reported to be involved in transcriptional stimulation through activation of the NF-κB pathway (15,30). We reported that the PKR/eIF-2α pathway was activated and that NF-κB translocation occurred during the OA-induced apoptosis (7,31). However, details of the mechanisms of OA-mediated expression and phosphorylation of IκB and NF-κB are still obscure. The relationship between IκB or NF-κB and PKR in apoptosis is also to be determined.

## Materials and methods

### Reagents

G418 Geneticin, cycloheximide (CHX), and anti-β-actin antibody were obtained from Sigma-Aldrich (St. Louis, MO, USA). α-modification of minimum essential medium (α-MEM), Opti-MEM, and pre-stained molecular weight markers were purchased from Gibco BRL (Grand Island, NY, USA). FuGene HD was from Roche (Indianapolis, IN, USA). Fetal bovine serum (FBS) was obtained from Equitech-Bio (Kerrville, TX, USA). Anti-phospho-eIF-2α (119A11) antibody was from Cell Signaling (Danvers, MA, USA). Anti-phospho-IκBα (Thr291), anti-PKR (M-515) and anti-NF-κB p65 (C-20) antibodies were from Santa Cruz Biotechnology (Santa Cruz, CA, USA). Antibody for IκBα (MAD-3) were obtained from BD Biosciences (San Jose, CA, USA). Plastic dishes were from Iwaki (Chiba, Japan). OA was purchased from Wako (Osaka, Japan).

### Cell culture and establishment of the PKR-K/R mutant MG63 cells

Human PKR cDNA and a PKR-K/R mutant cDNA (carrying a mutation of amino acid K→R at position 296) and their expression vector were kindly provided by Dr A. Hovanessian (Institute Pasteur, Paris, France) (32) and Dr T. Takizawa (Aichi Human Service Center, Aichi, Japan) (33), respectively. Human osteoblastic osteosarcoma cell line MG63 cells were obtained from the American Type Culture Collection (Rockville, MD, USA). The cells were cultured in α-MEM containing 10% (v/v) FBS and were maintained at 37°C in a humidified atmosphere of 5% CO_2_ and 95% air.

PKR-K/R cDNA was subcloned into pcDNA3.1-Flag (modified pcDNA3.1, Invitrogen, Carlsbad, CA, USA). Transfection of pcDNA3.1-Falg-PKR-K/R into MG63 cells was performed using FuGene HD reagents. Two *μ*g of pc-Flag and pc-Flag-PKR-K/R in 100 *μ*l Opti-MEM were mixed with 8 *μ*l FuGene HD reagent for 15 min at ambient temperature. The DNA-FuGene HD complex was then added into 35-mm dishes containing 4×10^5^ cells. The media were replaced at 24 h after transfection and the cells were subcultured in medium containing G418 Geneticin at a final concentration of 500 *μ*g/ml for 2 weeks. The media were replenished every 3 days. The drag-resistant colonies were selected and cloned. Cell modification was monitored by an Olympus IMT-2 phase-contrast microscope.

### SDS-PAGE and western blot analysis

MG63 cells and their PKR-K/R mutant cells were washed twice with PBS, scraped into lysate buffer containing 1 mM DTT, 1 mM PMSF, 1 *μ*g/ml leupeptin, 2 *μ*g/ml aprotinin, 5 mM EGTA and protein phosphatase inhibitor cocktail (Sigma-Aldrich) in phosphate-buffered saline (PBS). The lysates containing equal amounts of proteins were separated by 10% SDS-PAGE and transferred to PVDF membranes (Millipore, Bedford, MA, USA). The membranes were incubated for 2 h at ambient temperature in a blocking solution consisting of 5% non-fat skim milk in PBS containing 0.1% Tween-20 (PBS-Tween) and incubated overnight at 4°C with specific antibodies in PBS-Tween (diluted at 1:500 to 10,000). After the membranes had been washed 4 times within 30 min in PBS-Tween, they were incubated for 2 h at ambient temperature in PBS-Tween containing horseradish peroxidase-conjugated second antibodies (diluted at 1:5,000). The membranes were washed again as described above and the proteins recognized by the antibodies were visualized with an ECL detection kit (Pharmacia Biotech, Uppsala, Sweden) according to the manufacturer’s instuctions. To strip off the antibody the membrane was treated for 30 min at 50°C with 2% SDS and 0.35% 2-mercaptoehanol in 62.5 mM Tris-HCl (pH 6.8). The antibody-stripped membrane was then blocked again and re-incubated with other antibodies.

### RNA preparation, real-time PCR, and RT-PCR

MG63 cells and the PKR-K/R cells were cultured in 35-mm dishes (1.0×10^4^ cells/dish). Quantitative real-time PCR analysis was performed using SYBER Premix Ex taq Perfect Real-time (Takara Bio, Kyoto, Japan). The sequences of the primers used are as follows: IκBα forward, CACACGTGTCTACACTTAGCCTCTA; IκBα reverse, AATAGCCCTGGTAGGTAACTCTGTT; GAPDH forward, GACCCCTTCATTGACCTCAAC; GAPDH reverse, CTTCTCCATGGTGGTGAAGA. DNA amplification and detection was performed in the ABI PRISM 7500 (Perkin-Elmer Applied Biosystems, Foster City, CA, USA). PCR amplification (40 cycles) was performed following conditions: 95°C for 10 sec and 60°C for 34 sec. Standard curves were generated using 10-fold serial dilutions of genomic DNA. The concentrations of unknown samples were calculated by extrapolation from this standard curve and expression levels were normalized with GAPDH expression. The data were analyzed by Sequence Detection Software (SDS) vo1. 4 (Perkin-Elmer).

For RT-PCR analysis, total cellular RNA was extracted by using TRIzol (Invitrogen) and subjected to PCR using RT-PCR kit (Takara). The primers used for PCR were as indicated above. PCR reactions at 94°C for 30 sec, at 57°C for 30 sec and at 72°C for 30 sec were carried out for 32 cycles. The PCR products were separated by electrophoresis on 1.5% agarose gels and visualized by ethidium bromide staining with UV light illumination.

### DNA isolation and agarose gel electrophoresis

Purification of DNA from cultured cells was started by lysis of the cells in cold 10 mM Tris-HCl, pH 7.5, containing 1 mM EDTA and 0.5% Triton X-100. After lysis, debris was removed by centrifugation at 15,000 g for 20 min. DNAse-free RNAse (Sigma-Aldrich) was added to the lysates at a final concentration of 40 *μ*g/ml, and the lysates were then incubated with gentle shaking for 1 h at 37°C. Proteinase K (Sigma-Aldrich) was added to the RNAse-treated lysates at a final concentration of 40 *μ*g/ml. The lysates were further incubated for 1 h at 37°C with gentle shaking. DNA was precipitated with 2-propanol and sodium chloride overnight at −20°C. After centrifugation and drying, the DNA was dissolved in TE-buffer (10 mM Tris, pH 8.0, containing 1 mM EDTA). Agarose gel electrophoresis of DNA was performed by using 2.0% agarose gel containing 0.5 *μ*g/ml ethidium bromide. DNA markers (100 bp) (New England BioLabs, Ipswich, MA, USA) were run in the same gels. To visualize apoptotic alterations to DNA integrity, we observed the DNA bands on a UV transilluminator. Images were taken with a Polaroid DS-300 camera.

### Statistical analysis

All data are presented as mean ± SEM. Statistical analysis was performed using Student’s t-test. Results are representative examples of three or more independent experiments.

## Results

### Okadaic acid stimulated the expression of IκBα in MG63 cells

**Figure 1:**
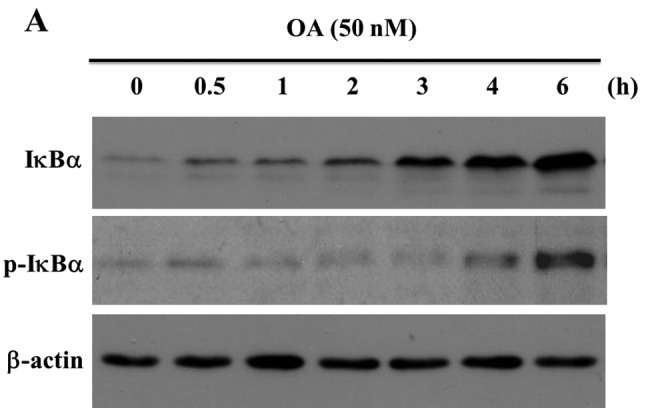

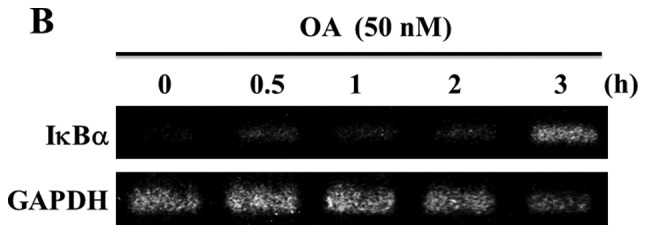
Expression of IκBα in the OA-treated cells. (A) After having reached the confluence, MG63 cells were treated for various time periods with 50 nM OA. Twelve *μ*g of each protein was separated on 10% of SDS-PAGE gels, transferred to PVDF membranes and incubated with antibodies specific for IκBα (upper panel) and anti-phospho-IκBα (middle panel), respectively. The bound antibodies were stripped off the membranes and re-incubated with anti-β-actin antibody as loading controls (lower panel). (B) Total cellular RNA was extracted from the OA-treated MG63 cells and subjected to RT-PCR for IκBα and GAPDH for the control.

Fig. 1A shows that the anti-IκBα antibody recognized a band corresponding to IκBα (36 kDa) in a sample prepared from the unstimulated MG63 cells. OA at 50 nM increased the expression of IκBα protein in a time-dependent manner (Fig. 1A, upper panel). The anti-phospho-IκBα (S32) antibody interacted with a band corresponding to IκBα and 50 nM OA increased the staining intensity of phosphorylated IκBα in MG63 cells (Fig. 1A, middle panel). The bound antibodies were stripped off the membranes and re-incubated with the anti-β-actin antibody as loading controls (Fig. 1A, lower panel). A band corresponding to the position of IκBα was not detected in the blots of the extracts incubated with the same dilution of normal rabbit serum (data not shown). We also analyzed the expression of IκBα mRNA. The result of RT-PCR shows that OA increased the expression of IκBα mRNA in MG63 cells (Fig. 1B).

**Figure 2:**
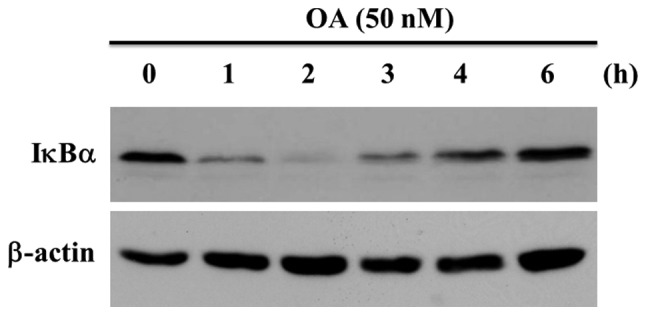
Degradation of IκBα in MG63 cells treated with OA. New protein synthesis in MG63 cells was inhibited by 100 ng/ml of CHX for 30 min. The cells were treated with 50 nM OA for various time courses as indicated (upper panel). The samples from the OA-treated cells were analyzed by western blotting with anti-IκBα antibody. The loading control done with β-actin is shown in the lower panel.

New protein synthesis in MG63 cells was inhibited by the treatment of 100 ng/ml of CHX for 30 min. The expression of IκBα protein was evaluated by western blot analysis. OA-treatment decreased the staining intensity of IκBα ≤2 h, at which time the staining level was minimum. After that the staining level increased in a time-dependent manner up to 6 h (Fig. 2). The amounts of IκBα decreased in MG63 cells treated with OA for 2 h in a dose-dependent fashion up to 100 nM (data not shown). These findings indicate that IκBα was degraded in MG63 cells with OA-treatment. The expression of β-actin was not changed with OA-treatment (Fig. 2).

### Expression of eIF-2α and PKR in the okadaic acid-treated cells

**Figure 3:**
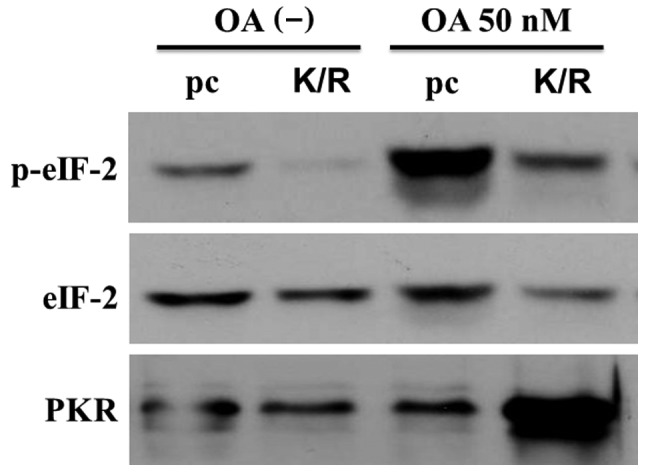
Expression of eIF-2α and PKR in the OA-treated cells. The established pc and PKR-K/R cells were cultured with 50 nM OA for 6 h. Phosphorylated eIF-2α and eIF-2α were detected with anti-phospho-eIF-2α and anti-eIF-2α antibodies, respectively. The cell lysates from the pc and PKR-K/R cells were analysed for the expression of PKR protein with anti-PKR antibody.

PKR functions via phosphorylation of IκBα (34,35) and OA increased the amounts of phosphorylated form of IκBα (Fig. 1). We considered that PKR might play an essential role in the phosphorylation of IκBα. We transfected MG63 cells with a catalytically inactive mutant of human PKR obtained by substituting Lys at 296 with Arg and established cells stably expressing dominant-negative PKR gene (PKR-K/R). To verify the PKR mutation, we analyzed the phosphorylation status of eIF-2α, the best-characterized substrate of PKR, by western blotting using an anti-phospho-eIF-2α antibody. Fig. 3 shows that the intensity of phosphorylated form of eIF-2α increased in the 50 nM OA-treated pcDNA-transfected MG63 cells (pc cells). However, the level of this form was low in the PKR-K/R cells treated with OA at the same conditions (Fig. 3). The amounts of eIF-2α did not differ between the control and PKR-K/R cells. OA increased the amount of PKR in the PKR-K/R cells whereas the expression levels of PKR were low in the OA-treated pc cells (Fig. 3). These data indicate that although PKR was overexpressed in PKR-K/R cells, functional PKR/eIF-2α pathway was inactivated by the transfection with the plasmid bearing the PKR dominant-negative mutation.

**Figure 4:**
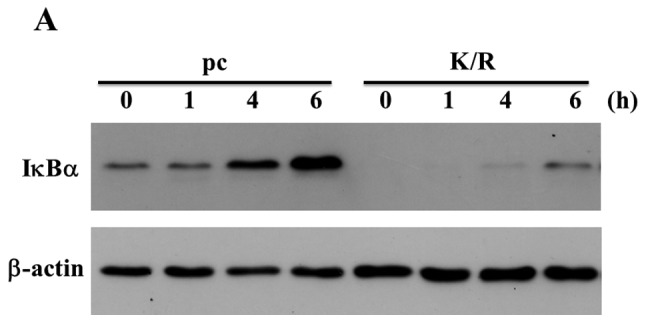

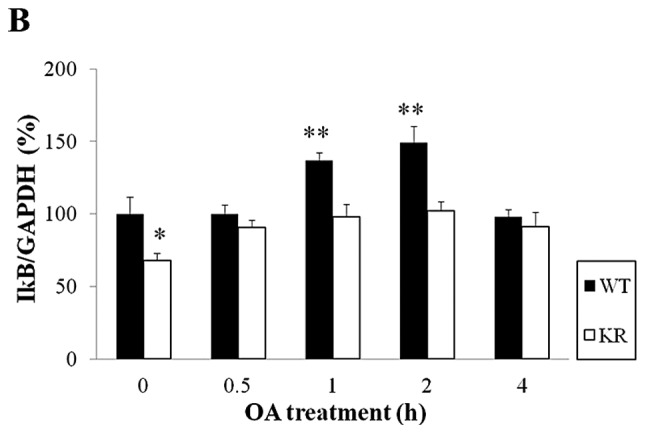
Expression of IκBα in pc and PKR-K/R cells. (A) The pc and PKR-K/R cells were treated for various time periods with 50 nM OA. The expression of IκBα was analyzed by western blotting. Loading controls were done with anti-β-actin antibody. (B) The expressions of IκBα mRNA in MG63 and PKR-K/R cells were analyzed by real-time PCR. Significant differences from the control cultures are indicated by asterisks; ^*^P<0.05; ^**^P<0.01; WT, MG63 cells; KR, PKR-K/R cells.

We treated pc and PKR-K/R cells with 50 nM OA for various time-points. Expression of IκBα was detected in the samples of the unstimulated pc cells whereas the expression levels of IκBα were low in the PKR-K/R cells (Fig. 4A). OA increased the IκBα expression in pc cells compared with that in PKR-K/R cells (Fig. 4A). The bound anti-IκBα antibody was stripped off and re-incubated with anti-β-actin antibody as a loading control (Fig. 4A). The result of real-time PCR shows that the expression of IκBα mRNA in MG63 cells was stimulated with 50 nM OA in a time-dependent manner (Fig. 4B). The expression of IκBα in the 50 nM OA-stimulated PKR-K/R cells was low compared with that of the wild-type MG63 cells (Fig. 4B).

### Regulation of NF-κB proteins in the cells treated with okadaic acid

**Figure 5:**
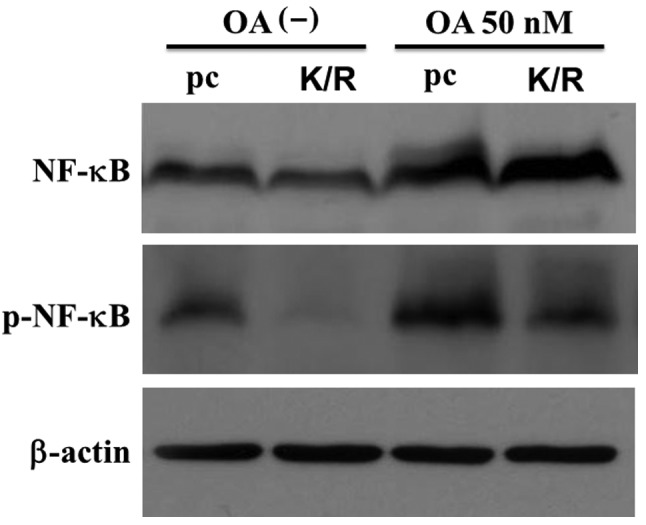
Western blot analysis of NF-κB in OA-treated cells. The pc and PKR-K/R cells were treated with 50 nM OA for 6 h and the cell lysates were prepared from each type of culture. Twelve microgram of each sample was separated on a 10% of SDS-PAGE and transferred to PVDF membranes. Each membrane was then incubated with anti-p65NF-κB (NF-κB) and anti-phospho-Ser536 NF-κB (p-NF-κB) antibodies. The membrane antibody was stripped off and re-incubated with anti-β-actin antibody as a loading control.

To determine the expression of NF-κB in pc and PKR-K/R cells whole cell lysates prepared from the 50 nM OA-stimulated cells were analyzed by western blotting with an antibody against p65NF-κB. The anti-p65NF-κB antibody interacted with a major band having an estimated molecular weight of 65 kDa (Fig. 5, upper panel). This antibody also recognized a more slowly migrating band in the OA-treated pc cells. The levels of slowly migrated bands in the PKR-K/R cells were low compared with that in the pc cells (Fig. 5). These slower migrated bands were not detected in the extracts prepared from the unstimulated cells. The slowly migrated band was a phosphorylated form of NF-κB (31). Fig. 5 also shows that the anti-phospho-Ser536 p65NF-κB antibody interacted with a 65-kDa band and OA increased the staining intensity of the band in pc and PKR-K/R cells. However, the staining intensity was higher in pc cells than that in the PKR-K/R cells (Fig. 5, middle panel). The bound antibody was stripped off the membrane and re-incubated with anti-β-actin antibody for the loading controls (Fig. 5, lower panel).

### Apoptosis in MG63 and PKR-K/R cells

**Figure 6:**
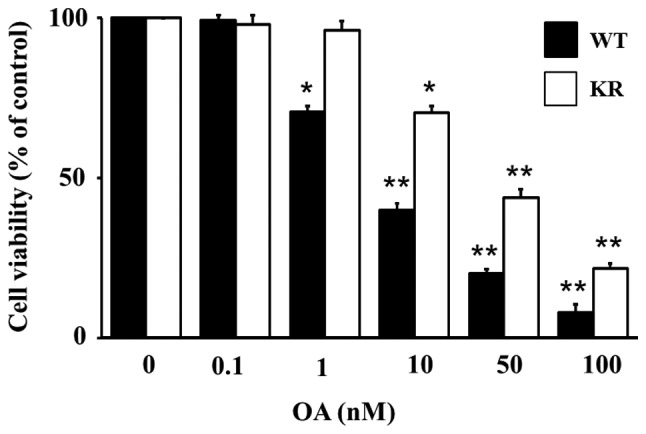
Effects of OA on cell viability in MG63and PKR-K/R cells. MG63 (WT) or PKR-K/R (KR) cells grown in 96-well plates were treated for 24 h with various concentrations of OA and the cell viability was determined by the WST-8 assay. The activity was compared to the control well of the same cell line and results are expressed as a percentage of the control (means ± SEM) (N=7). The absorbance at 405 nm of the control cultures for MG63 or PKR-K/R were 1.089±0.082 or 1.104±0.088, respectively. Significant differences from the control cultures are indicated by asterisks; ^*^P<0.05; ^**^P<0.01 (Student’s t-test).

In our previous study, OA induced apoptosis in MG63 cells (7). We examined whether OA could induce apoptosis in the PKR-K/R cells. To quantify the OA-induced cytotoxicity in MG63 and PKR-K/R cells, the cells were treated with various concentrations of OA for 24 h and the cell viability was measured by the WST-8 assay. Fig. 6 shows that OA decreased the cell viability in MG63 cells in a dose-dependent manner up to 100 nM. OA at 10 nM decreased the cell viability to ∼40% that of the control cells, whereas the viability of 50 nM OA-treated cells was 20% that of the control cultures (Fig. 6). OA also decreased the cell viability in PKR-K/R cells (Fig. 6). However, the level of cell viability was higher in the PKR-K/R cells compared with that in the MG63 cells (Fig. 6). The cell viability of PKR-K/R cells treated with 50 nM OA was 40% that of the control cells (Fig. 6).

**Figure 7:**
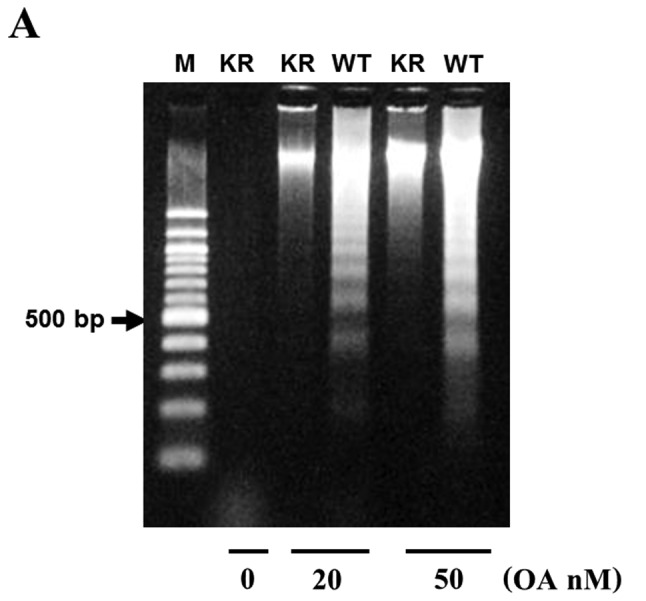

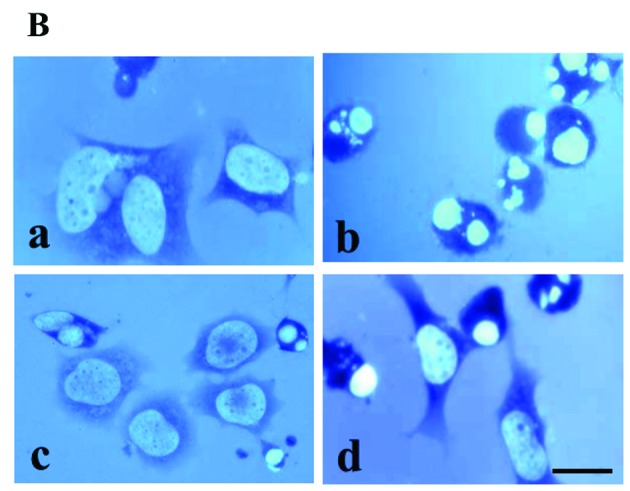
DNA ladder formation (A) and nuclear fragmentation (B) in MG63 and PKR-K/R cells treated with OA. (A) Confluent cells were exposed to OA with low (20 nM) or high (50 nM) concentrations for 48 h. Extracted DNA was electrophoresed through an agarose gel and stained with ethidium bromide. M, standard DNA markers; Arrow indicates 500 bp. (B) Confluent MG63 and PKR-K/R cells were incubated with 50 nM OA for 48 h. The cells were stained with Hoechst 33342 and evaluated the nuclear fragmentation and condensation of chromatin. a, control MG63 cells; b, 50 nM OA-treated MG63 cells; c, control PKR-K/R cells; d, 50 nM OA-treated PKR-K/R cells. Bar represents 10 *μ*m.

To determine if the OA-induced cell viability was due to apoptosis, we looked for the presence of nuclear fragmentation in MG63 and PKR-K/R cells treated with a low (20 nM) or higher (50 nM) concentrations of OA for 48 h. The extracted DNA was analyzed by agarose gel electrophoresis and stained with ethidium bromide. In the 50 nM OA-treated MG63 cells, a DNA fragmentation pattern forming a ladder of multiples of 185–200 bp was observed (Fig. 7A). However, DNA laddering pattern was minimum in the PKR-K/R cells treated with the same concentration of OA. OA at 20 nM also stimulated the DNA laddering pattern in MG63 cells however the same concentration of OA did not induce DNA laddering in the PKR-K/R cells (Fig. 7A). We also evaluated the nuclear fragmentation and condensation of chromatin in MG63 and PKR-K/R cells by Hoechst staining. The control cells did not show any apoptotic features in MG63 cells and PKR-K/R cells (Fig. 7Ba and c). In the 50 nM OA-treated MG63 cells nucleic acid staining with Hoechst 33342 exhibited typical apoptotic nuclei, which had highly fluorescent condensed chromatin structures (Fig. 7Bb). However, in the PKR-K/R cells, the number of the apoptotic cells significantly decreased, although the cells still manifested apoptotic features (Fig. 7Bd).

## Discussion

We transfected a human cDNA having the amino acid lysine at 296 replaced with Arginine in the catalytic domain of PKR into human osteoblastic MG63 cells and established the stable cell lines that express mutant gene construct (PKRK/R cells). Phosphorylation of eIF-2α was not detected in the PKR-K/R cells whereas strong phosphorylation of eIF-2α occurred in pcDNA-transfected MG63 cells. Because eIF-2α is a substrate for PKR (6,7), the mutant cells we established have a PKR dominant-negative characteristics. OA stimulated the phosphorylation of eIF-2α in MG63 cells, however, phosphorylation of eIF-2α in PKR-K/R cells was not stimulated with the OA-treatment.

We explored the effects of OA on the expression and phosphorylation of IκBα and p65NF-κB in MG63 and PKR-K/R cells. During the OA-treatment, the expression and phosphorylation of IκBα increased in MG63 cells. IκBα was degraded upon OA treatment in MG63 cells. We previously demonstrated that IκBα was phosphorylated on tyrosine residues by the OA-treatment (36). In the present study IκBα was phosphorylated at least on serine residues at 32 position, because the anti-phospho IκBα (Ser32) recognized the phosphorylated form of IκBα. We also detected the phosphorylation at Ser36 in MG63 cells (data not shown).

OA is one of the many stimuli activating NF-κB in the cultured cells. It has been reported that OA increased the phosphorylation of cellular proteins (24). We previously reported that OA induced activation of PKR/eIF-2α, nuclear translocation of p65NF-κB and apoptosis in MG63 cells (5,7,31). In human neutrophils and HL-60 cells, OA and orthovanadate, an inhibitor of tyrosine phosphatase, stimulated the activation of NF-κB and rapid degradation of IκBα (37). The NF-κB activation was caused by the OA-induced inhibition of PKCδ and IKK phosphatases or by the OA-induced activation of ERK1, a member of the MAP kinase family (37). These reports indicate that the phosphorylation and degradation of IκBα was influenced by OA-sensitive phosphatases. However, it was reported that OA-induced activation of NF-κB did not depend on the inhibitor properties of OA but rather on the production of reactive oxygen intermediates (38). The phosphorylation of IκBα preceding its degradation occurs on the Ser32 and 36 residues, making it a subject to degradation by proteosomes. These findings consist with our present results. Degradation of IκBα liberates the NF-κB complex, which is able to migrate to the nucleus and to activate gene expression (39). In our previous study we demonstrated that 100 nM OA-treatment induced IκBα phosphorylation without causing its degradation. This phosphorylation appears to occur on a tyrosine residue because anti-phospho-tyrosine antibody bound to the samples immunoprecipitated with the anti-IκBα antibody (36). This finding confirms an earlier one that tyrosine phosphorylation of IκBα induced NF-κB activation without its degradation (40). In the present study, we demonstrated that 50 nM OA-treatment induced phosphorylation of IκBα on Ser32 residue and degradation of IκBα. The discrepancy of the results derived from the use of CHX to block the new protein synthesis in the present study.

PKR is an interferon-induced protein, initially identified and characterized as a translational inhibitor in an antiviral pathway regulated by interferon (1,2). It was reported that PKR could function as a signal transducer for mediating transcriptional activation in response to dsRNA via its ability to phosphorylate IκBα, resulting in the activation of NF-κB (41). The relationships between PKR and IκBα has not been reported previously. To provide a further insight into the role of phosphorylation of IκBα in PKR pathway, we used PKR-K/R cells stably expressing the dominant-negative PKR. PKR was earlier found to be associated with IKK complex, where its major contribution appears to be activation of NF-κB (35,41). In our previous study, we demonstrated that the mutation of PKR kinase resulted in the basal translocation of p65NF-κB into the nucleus in the unstimulated cells (7,31). This translocation was accompanied by the degradation of IκBα. These results indicate that functional PKR is necessary for the cytosolic localization of NF-κB and for the phosphorylation of IκBα in the OA-stimulated cells. Expression of the PKR mutation is associated with enhanced levels of NF-κB DNA-binding and transcriptional activities compared with those of the control cells (42), further supporting our present results. Serine phosphorylation of IκBα and its degradation did not require PKR kinase activity (41). We demonstrated that IκBα was degraded without phosphorylation in PKR-K/R cells suggesting that the kinase activity of PKR is required for the phosphorylation of IκBα. PKR has Ser/Thr kinase activity; however, it also phosphorylates tyrosine residue in place of Ser51 in eIF-2α (42). The relationship between kinase activity of PKR and other kinases, such as c-Src, remains to be examined.

PKR is required to osteoblast differentiation, osteoclast formation and chondrogenesis (43–46). Phosphorylation of IκBα plays important roles in osteoclastogenesis, osteoclast recruitment and osteolysis (45,47,48). We previously reported that OA induced apoptosis in osteoblastic cells (7,27–31). During OA-induced apoptosis, the PKR/eIF-2α pathway was activated, that activation was followed by the inhibition of protein synthesis (7). Although the detailed functions of PKR activity in osteoblastic apoptosis remain unclear, it might be possible that PKR mediates the OA-induced apoptosis in MG63 cells by phosphorylating IκBα and causing p65NF-κB translocation. It still remains to be determined whether an OA-sensitive phosphatase could regulate this pathway.
